# Bleeding in Critically Ill Children—Review of Literature, Knowledge Gaps, and Suggestions for Future Investigation

**DOI:** 10.3389/fped.2021.611680

**Published:** 2021-01-27

**Authors:** Adi Avniel Aran, Oliver Karam, Marianne E. Nellis

**Affiliations:** ^1^Pediatric Cardiac Critical Care Division, Hadassah University Medical Center, Jerusalem, Israel; ^2^Division of Pediatric Critical Care Medicine, Children's Hospital of Richmond at VCU, Richmond, VA, United States; ^3^Pediatric Critical Care Medicine, NY Presbyterian Hospital–Weill Cornell Medicine, New York, NY, United States

**Keywords:** bleeding, hemorrhage, scale, epidemiology, critical ill children

## Abstract

Clinically significant bleeding complicates up to 20% of admissions to the intensive care unit in adults and is associated with severe physiologic derangements, requirement for significant interventions and worse outcome. There is a paucity of published data on bleeding in critically ill children. In this manuscript, we will provide an overview of the epidemiology and characteristics of bleeding in critically ill children, address the association between bleeding and clinical outcomes, describe the current definitions of bleeding and their respective limitations, and finally provide an overview of current knowledge gaps and suggested areas for future research.

## Introduction

Clinically relevant bleeding can commonly affect critically ill patients, either as an admission diagnosis or as a complication of underlying critical disease. In one large, prospective cohort of critically ill adults, approximately one-fifth had significant bleeding during their admission to the ICU (1). One-tenth of critically ill children were reported to be bleeding on admission in a retrospective study in the PICU. In addition, a similar number developed clinically relevant bleeding during their stay (2).

Physiologically, clinically relevant bleeding (CRB) may lead to reduced oxygen delivery in two ways: (1) decreased blood oxygen content due to lower red blood cell mass and (2) decreased cardiac output due to hypovolemia. Poorer clinical outcomes have been associated with bleeding in subpopulations of critically ill patients. These include increased mortality in bleeding adults with acute coronary syndrome (3) and in those following bone marrow transplant (4). Adults who undergo cardiac surgery and have significant post-operative bleeding are more likely to be mechanically ventilated longer and stay longer periods in the ICU (5). Likewise, poorer clinical outcomes have been observed in critically ill children who bleed including more exposure to vasoactive medications and longer lengths of stay in both the ICU and the hospital as compared to those who do not bleed (2). Nevertheless, until recently, there was no published definition accurately describing the severity of bleeding in critically ill children. We lack large epidemiologic studies documenting prevalence of CRB, as well as risk factors and effective prevention.

In this review, we will provide an overview of the existing evidence regarding the epidemiology and characteristics of bleeding in critically ill children, describe the association between bleeding and clinical outcomes, discuss the current definitions of bleeding and their respective limitations, and, in doing so, provide an overview of current knowledge gaps and suggestions for areas of future research.

## Epidemiology of Bleeding in The General PICU Population: Incidence, Identified Risk Factors, and Outcome

Despite the potential morbidity of CRB for critically ill children, less than a handful of studies depict the epidemiology and potential risk factors of CRB in the general Pediatric Intensive Care Unit (PICU) population. Current epidemiologic studies are typically limited to certain subsets of critically ill patients, such as those undergoing cardiac repair with cardiopulmonary bypass (6–8), or to specific anatomical locations of bleeding, such as chest tubes (6–8) or the gastrointestinal tract (9–11). None of the published studies to date used a validated pediatric definition of CRB and all of the definitions varied.

In a single-center retrospective cohort study conducted over 6 months in a tertiary care children's hospital, Moorehead et al. reported CRB occurring in 9% (19/214) of their patients (12). The authors defined CRB in relation to hematoma formation, the development of hemorrhagic shock, a defined drop in hemoglobin concentration, the cessation of anticoagulant medication due to bleeding CRB, or fluid resuscitation with either crystalloid or red blood cell (RBC) transfusions to control bleeding. Thrombocytopenia (platelet count < 100 × 10^9^/L), mechanical ventilation, the use of either antibiotic or antacid medications, and cardiac surgery were patient factors that were each associated with increased risk of CRB. The study was limited by significant disagreement between those reviewing the charts as to which patients met their defined criteria for CRB (kappa statistic = 0.26).

To follow up on this study and account for its limitations, Moorehead et al. applied their definition of CRB to a prospective cohort of critically ill children (13). In particular, they reported on the associations between laboratory values and CRB. Two hundred and thirty-four children were enrolled and a similar incidence of CRB was reported. There was a suggestion of decreasing platelet count being associated with increased bleeding. However, the association was not significant when accounting for confounders. Irregularities in other measures of coagulation, such as international normalized ratio (INR) and activated partial thromboplastin time (aPTT), were not associated with increased bleeding. The study was limited by incomplete laboratory data for each patient enrolled.

In a cohort study in a single-center PICU, White et al. reported CRB in 9% (37/405) of the children admitted to the PICU (14). They modified a previously developed tool from the International Society on Thrombosis and Hemostasis (ISTH) and characterized bleeding as either major, clinically relevant non-major, or minor. The majority of children, ~80%, had only one bleeding event. Children receiving invasive mechanical ventilation or stress ulcer prophylaxis were more likely to have CRB. Though mortality was no different, those with CRB had longer lengths of stay both in the ICU and the hospital as a whole.

Pinto et al. used the same bleeding tool (known as the ISTH-BAT) to assess the epidemiology of CRB in critically ill adolescents with increased risk for venous thromboembolism (VTE) (15). The frequency of CRB was 30% (95% CI, 20–40%). The high incidence of CRB reported in this study probably reflects the inclusion of bleeding events occurring on the day of admission that were excluded from the previous studies discussed. As a result, the proportion of surgical and trauma patients in this study was also higher. CRB was more commonly seen in adolescents with VTE, likely reflecting increased risk of bleeding due to anticoagulation. Younger age, higher predicted risk of mortality score, and admission for trauma or surgery were also associated with increased risk for CRB.

In conclusion, there is limited data on the epidemiology of CRB in the general PICU population. Reported incidence of bleeding in single center studies was 9–30%, depending on inclusion criteria and based on different bleeding assessment scales. Risk factors identified were admission due to cardiac surgery or trauma, young age, mechanical ventilation, receipt of antacid medications and thrombocytopenia. Patients with CRB had longer ventilation days, increased requirement for vasoactive drugs and blood products, and longer PICU length of stay.

## Bleeding in Selected High Risk Populations of Critically Ill Infants and Children: Characteristics and Etiologies

Several subsets of critically ill children may be particularly prone to bleeding complications. ICU diagnoses associated with increased frequency of bleeding include trauma, cardiac surgery, orthopedic surgery, hematologic malignancies, sepsis, respiratory failure (requiring invasive mechanical ventilation), renal failure requiring renal replacement therapy, and extracorporeal membrane oxygenation (ECMO) (16, 17).

As seen in epidemiologic studies, younger age is associated with an increased risk of bleeding. This may be secondary to the characteristics of the normal development of the coagulation system in infancy. Prior to 6 months of age, infants have lower concentrations of vitamin K–dependent pro-coagulant clotting factors and reduced thrombin potential (18), ~70% of the levels as compared to adults. Prothrombin levels are also significantly lower as compared with that in adults (19). It is unclear whether these lower levels are actually associated with more episodes of CRB.

In addition to patient characteristics, there are several physiologic derangements that occur during critical illness that may worsen bleeding. These include hypothermia, acidosis, excessive fibrinolysis, and consumption of coagulation factors and platelets (20). The activity of coagulation factors, as well as necessary enzymes, are inhibited during these physiologic states (21).

We will further discuss selected groups of PICU patients who may be particularly prone to medical and surgical CRB (see Table 1).

**Table 1:** Medical and surgical conditions associated with clinical relevant bleeding in children.

**Medical condition**	**Surgical condition**
Renal failure and hemodialysis	Spinal surgery
Gastrointestinal bleeding	Cardiac surgery
Sepsis and disseminated intravascular coagulation	Extracorporeal membrane oxygenation
Idiopathic pulmonary hemorrhage	
Central nervous system bleeding	

## Medical Bleeding

### Renal Failure and Hemodialysis

Acute kidney injury (AKI) is a common complication of critical illness, affecting almost 30% of the children admitted to pediatric intensive care units (22). Many of these patients develop significant enough AKI to require dialysis, often secondary to fluid overload. In prospective studies in adults, bleeding has been reported in 14–50% of patients with dialysis-dependent AKI, and is described as a major risk factor for mortality (23, 24). Similar to previously mentioned populations, there is very little published data concerning dialysis-dependent AKI and bleeding in the pediatric population.

Kreuzer et al. published a retrospective study of bleeding in nearly 200 children with dialysis-dependent acute kidney injury (25). Slightly over a quarter of the children had bleeding events with 62% of the episodes considered life-threatening. Several factors were independently associated with a higher risk of bleeding including the underlying primary disease leading to AKI, presence of multiorgan dysfunction syndrome (MODS), recent surgery or bleeding prior to the onset of AKI, and hemodialysis or continuous veno-venous hemofiltration (as compared to peritoneal dialysis). In addition, the presence of bleeding was independently associated with mortality in this patient population.

Patients with uremia may be more prone to bleed due to several factors including platelet dysfunction, vitamin K deficiency, and/or slower clearance of unfractionated or low molecular weight heparin which is usually given during dialysis (26). Children with dialysis-dependent AKI are an understudied population in hemostatic research.

### Upper Gastrointestinal Bleeding

Although perceived as a prevalent complication in the PICU, there is limited data on the incidence, risk factors and outcome of upper gastrointestinal bleeding in critically ill children. In a single-center prospective study, Chaibou et al. reported upper GI bleeding in 10% (103/1006) of their cohort, with only two percent (16/1006) considered CRB (9). CRB was adjudicated by three authors and had to be associated with significant hemoglobin drop, RBC transfusion, hypotension, development of MODS, surgery, or death. Three independent risk factors for clinically relevant upper GI bleeding were respiratory failure, coagulopathy, and pediatric risk of mortality score >10.

In a more recently published retrospective study, Sahin et al. reviewed records of 136 eligible children consecutively admitted to their tertiary care PICU. The overall incidence of upper GI bleeding was 15%. However, the study was limited by small sample size in that only one child developed what was considered significant bleeding. Approximately 30% of the children who received antacid prophylaxis developed any upper GI bleeding. Mechanical ventilation, trauma, coagulopathy, thrombocytopenia, higher severity score at admission, renal and hepatic failure, hypotension, heart failure, and arrhythmia were found to be associated with the development of any upper GI bleeding (27).

### Sepsis and Disseminated Intravascular Coagulation (DIC)

Sepsis is commonly associated with the development of thrombocytopenia, hypofibrinogenemia, and prolongation of prothrombin time (28) which may manifest as multisite bleeding including petechiae, hematomas, and pulmonary hemorrhage. However, sepsis can also trigger the extrinsic coagulation cascade and result in microthrombi (29). In a recently published small cohort of children with septic shock, the incidence of DIC was estimated to be 42–66% (depending on the definition of DIC applied) (30). Though the presence of DIC in children with septic shock has been associated with higher mortality (31), the incidence of bleeding vs. clotting with DIC in these children is not clear. The incidence of bleeding and thrombotic complications in one cohort of Thai children with gram negative sepsis and DIC was 59 and 20%, respectively (32).

### Idiopathic Pulmonary Hemorrhage (IPH)

Bleeding into the lower respiratory tract can occur acutely (diffuse alveolar hemorrhage) or more chronically (idiopathic pulmonary hemosiderosis). IPH is a diagnosis of exclusion and other causes, such as gastrointestinal bleeding, cows' milk allergy, cystic fibrosis, or vasculitis must be excluded. The incidence of both acute and chronic IPH is rare. In a 10-years retrospective study of infants at Boston Children's Hospital, only 157 cases were identified (33). Those infants most commonly had associated congenital heart disease, chronic lung disease of prematurity, congenital or acquired lung disorders, or congenital or acquired coagulopathies.

### Central Nervous System (CNS) Bleeding

The etiologies of bleeding into the central nervous system of children is varied and includes trauma (both accidental and non-accidental), prematurity, underlying congenital or acquired coagulopathies, idiopathic thrombocytopenia purpura, rupture of arteriovenous aneurysms, or hemorrhage into CNS tumors. Given the diverse pathologies, the incidence of and risk factors for CNS bleeding in pediatrics varies.

## Surgical Bleeding

### Pediatric Spinal Surgery

Children undergoing spinal surgery, most often correction of scoliosis, are at high risk of bleeding given the length of surgery and the surgical technique required (involving bone dissection and possible disruption of vertebral blood vessels) (34). It is not uncommon for children to lose their entire circulating blood volume. In a large database analysis of blood utilization during spinal surgery from 2000 to 2009, nearly one-third of children required RBC transfusion (35). Survey studies have suggested that the rate of massive bleeding has decreased over time (36), though these studies may be confounded by response bias. The use of antifbrinolytics during surgery (such as tranexamic acid or aminocaproic acid) may have driven the trend of decreased bleeding and decreased post-operative blood product exposure in children undergoing spinal repair (37).

### Cardiac Surgery

Children undergoing cardiac repair are also at significant risk of perioperative and post-operative bleeding. Several pre-operative factors may contribute to the bleeding risk. These repairs are often performed during the first year of life. At baseline, infants with congenital heart disease may have lower levels of clotting factors (38). Additionally, cyanotic heart disease has been associated with thrombocytopenia and platelet dysfunction (39–41). Intra-operative blood loss may be pronounced due to coagulopathy induced by the cardiopulmonary bypass circuit, as well as induced hypothermia, use of high dose anticoagulation, and hemodilution (42–47).

Post-operative bleeding is a common complication in pediatric patients undergoing cardiopulmonary bypass (CPB) and is associated with increased morbidity and mortality (46–48). The receipt of post-operative blood products is independently associated with longer lengths of mechanical ventilation and length of PICU stay (49). Post-operative bleeding may be predicted by several factors including thrombocytopenia and hypofibrinogenemia (6, 8).

The Network for the Advancement of Patient Blood Management, Haemostasis, and Thrombosis (NATA) recently published guidelines to minimize blood product exposure in these vulnerable children (50). Their recommendations include the diagnosis and treatment of peri-operative anemia, prophylactic use of either tranexamic acid or aminocaproic acid in the operating room, priming of the cardiopulmonary bypass pump with colloid (as opposed to crystalloid), use of modified ultrafiltration and cell salvage during bypass, and the development of institution-specific transfusion algorithms.

Outside of the post-operative period, critically ill pediatric patients with heart disease can also experience CRB. Patients receiving any form of anti-coagulation (e.g., unfractionated heparin [UFH], low molecular weight heparin [LMWH], and warfarin) are at risk for bleeding secondary to decreased thrombin generation. Acquired von Willebrand disease (vWD) should be considered in patients with excessive mucosal bleeding and the presence of a vascular lesion that creates high shear stress, such as aortic stenosis or a left ventricular assist device (VAD). Definitive treatment of acquired vWD is the correction of the underlying cardiac abnormality, but this is not always possible. Alternatively, treatment with a von Willebrand factor (vWF) containing replacement product can be considered (51).

### Extracorporeal Membrane Oxygenation (ECMO)

ECMO may be used to rescue children with profound respiratory and/or heart failure and its use in pediatrics is increasing (52). Like CPB, ECMO is associated with bleeding and is multifactorial secondary to hemodilution, circuit induced coagulopathy, and platelet dysfunction, among other causes. Bleeding and thrombosis, including intracranial hemorrhage and stroke, are frequent and challenging complications in the management of pediatric ECMO patients and associated with increased risk for mortality (53–55). Dalton et al. conducted the first multicenter prospective observational study of children on ECMO examining the incidence, risk factors and associated outcomes of bleeding and clotting (56). Bleeding complications were defined as those requiring RBC transfusion and those occurring in the intracranial space. In a cohort of 514 children followed over 28 days, the incidence of bleeding was 70% (16% of which were intracranial) and the incidence of thrombosis was 38%. Age, indication for ECMO, organ failure at the time of ECMO initiation, and clinical site were all independent predictors of bleeding. Two of three discharge functional status scales were worse in patients who experienced bleeding in comparison to those who did not.

We conducted a secondary analysis of this cohort to evaluate the association between quantifiable bleeding (as measured from chest tubes) and clinical outcomes in children supported by ECMO (57). Over half of the patients had bleeding documented from the chest tube. The median amount of bleeding from the entire population over the course of ECMO was slightly over 120 mL/kg [interquartile range 47–319 mL/kg]. A “dose-dependent” response was observed in the independent association between amount of bleeding and mortality, as well as PICU length of stay. Whereas, some coagulation measures, such as fibrinogen level, were independently associated with chest tube bleeding, other measures, such as platelet count, were not.

## Bleeding Scales Applicable to Critically Ill Children—Where are We Today?

As we portrayed thus far, bleeding represents a common complication in the critically ill child, with significant morbidity. In addition, the interventions used to treat CRB, such as blood product, medication administration, and/or surgical procedures, may also be associated with worsened outcomes. Research is needed to guide the clinician as to the correct situations when interventions are needed to treat CRB. However, a standardized definition is needed to conduct these studies.

There are certainly common elements within many of the currently published definitions of bleeding. Definitions often include the amount and site of bleeding (58–61), physiologic or laboratory effects of bleeding (60, 62) and the need for transfusion or other medical intervention to treat the bleeding (59–62). The latter criteria, the need for transfusion or intervention, can add significant variability to the measurement of bleeding incidence as these measures are subjective and may be based on clinician preference. Most definitions have been developed to be specifically applicable to certain patient populations including those with cancer (58, 59, 62), receiving anticoagulation (60) or with inherited bleeding disorders (61). These do not address the specific risk factors for and characteristics of bleeding in critically ill children (e.g., bleeding from an endotracheal tube). Until quite recently, the HEmorrhage MEasurement (HEME) score was the only diagnostic criteria developed to evaluate CRB in critically ill patients (1) and included specific physiologic (such as heart rate) and laboratory (such as hemoglobin) measures. However, it was developed for critically ill adults, and therefore cannot be applied to critically ill children due to different age specific norms.

We conducted a systematic review in order to identify current bleeding scales and their validity to assess bleeding in critically ill children (63). Studies were included if they contained a bleeding score, bleeding measurement tool or clinical measurement of hemorrhage. Of the 18 studies identified, only two described critically ill children. As with previously mentioned scales in adults, the majority of identified bleeding scales included the need for medical or surgical intervention as a part of the definition. In addition, the majority of scales did not report measures of reliability or validation to clinical outcomes. This review highlighted the need for the development of an objective, validated, operational definition that included characteristics of bleeding particular to critically ill children. The National Institute of Health, National Heart, Lung and Blood Institute (NHLBI) and the Biomedical Excellence for Safer Transfusion (BEST) Collaborative recognized this need as well and identified the development of a validated bleeding measurement scale in critically ill children (as well as other vulnerable patient populations) as a high priority (64, 65).

As the next step in the development of a definition of bleeding in critically ill children, we performed a web-based international survey to qualify the clinical significance of different bleeding characteristics in critically ill children (66). We identified four domains of bleeding (site, amount, severity, and progression) and asked pediatric intensivists what they considered clinically relevant vs. irrelevant. Two hundred and twenty-five pediatric intensivists from 16 countries responded. They identified the following characteristics as clinically relevant: bleeding in critical locations (such as the pericardium, pleural space, central nervous system, and lungs), bleeding requiring interventions (such as chest tube insertion or the administration of blood products), bleeding leading to physiologic repercussions (such as organ failure or hypoperfusion); and bleeding lasting more than 6 h. In particular, quantifiable bleeding of more than 5 mL/kg/h for more than 1 h was frequently considered clinically relevant. In addition, providers identified several specific clinical scenarios of bleeding as being clinically irrelevant, including streaks of blood in the endotracheal tube and non-coalescing petechiae.

Based on these identified and characterized bleeding elements, we conducted a Delphi consensus process with over 30 multidisciplinary experts in bleeding and hemostasis in order to design a standard definition for CRB in critically ill children (67). The results of the process, the Bleeding Assessment Scale in Critically Ill Children (BASIC), are presented in Table 2. The experts divided bleeding into severe, moderate and minimal and described clinical characteristics of each category. Qualifiers were made to describe fatal and progressive bleeding. In order to incorporate clinically relevant physiologic consequences of bleeding into the scale, the experts used organ dysfunction as measured by the Pediatric Logistic Organ Dysfunction (PELOD)-2 score (68). Given the PELOD-2 scale includes physiologic derangements within nearly every system, the experts chose not to include each and every possible location of bleeding within the definition. Instead, they only included three sites (intraspinal, intraorbital and intraarticular) that though rare, would not be reflected in the PELOD-2 scoring system. In addition, changes in heart rate, blood pressure and hemoglobin were described by proportional differences, as opposed to whole numbers, in order to account for age-related standards. Lastly, the experts chose to exclude clinical interventions within the definition, based on both the subjectivity of clinical decision-making, as well as retaining the ability of the use of the definition in studies to assess the value of such interventions.

**Table 2:** Consensus definition of clinically relevant bleeding (59).

**BLEEDING ASSESSMENT SCALE IN CRITICALLY ILL CHILDREN**
**Any of the following criteria define severe bleeding:**
•Bleeding that leads to at least one organ dysfunction, using PELOD-2 score criteria of organ dysfunction, within 24 h of the previous assessment (if there is no previous assessment, the baseline results are presumed to be normal). The organ dysfunction should be associated with the bleeding, in absence of other causes.
•Bleeding that leads to hemodynamic instability, defined as an increase in HR by >20% from baseline or a decrease in BP by >20% from baseline. The hemodynamic instability should be associated with the bleeding, in absence of other causes.
•Bleeding leading to a drop in hemoglobin >20% within 24 h. The drop in hemoglobin should be associated with the bleeding, in absence of other causes.
•Quantifiable bleeding ≥5 mL/kg/h for ≥1 h (e.g., chest tube, drain).
•Intraspinal bleeding leading to loss of neurologic function below the lesion, non-traumatic intra-articular bleeding leading to decreased range of movement, or intraocular bleeding leading to impaired vision.
**All the following criteria must be present to define moderate bleeding:**
•Bleeding more than minimal bleeding but without criteria for severe bleeding.
•Bleeding not leading to organ dysfunction, as measured by the PELOD-2 score.
•Bleeding not leading to hemodynamic instability, i.e., change in HR >20% or drop of BP <20% from baseline.
•Bleeding leading to a drop in hemoglobin ≤20%.
•Quantifiable bleeding ≥1 but <5 mL/kg/h.
**Any of the following criteria define minimal bleeding:**
•Streaks of blood in ETT or during suctioning only.
•Streaks of blood in nasogastric tube.
•Macroscopic hematuria, or ≤1+ (≤1+) RBCs present on urine dipstick if available.
•Subcutaneous bleeding (including hematoma and petechiae) <5 cm (2 inches) in diameter.
•Quantifiable bleeding <1 mL/kg/h (e.g., chest tube, drain).
•Bloody dressings required to be changed no less than each 6 h, or weighing no >1 mL/kg/h if weighed, due to slow saturation.
**Progressive bleeding**: progressive bleeding is bleeding that either progresses to a higher severity category (e.g., from minimal to moderate bleeding, or from moderate to severe bleeding) or to a higher number of criteria within the same category (e.g., hemodynamic instability progressing to organ failure, or streaks of blood in the ETT and subsequent slightly blood-tinged urine).
**Fatal bleeding**: bleeding that is the direct cause leading to death will be characterized as fatal bleeding.

*BP, blood pressure; ETT, endotracheal tube; HR, heart rate; PELOD, Pediatric Logistic Organ Dysfunction*.

The BASIC definition underwent preliminary internal validity testing as it was applied to 40 critically ill children with 46 distinct bleeding episodes in a single center (67). When comparing the application of the definition to describe the bleeding episodes by two independent observers, there was substantial inter-rater reliability [kappa statistic 0.74 (95% CI, 0.6–0.9)]. The BASIC definition represents the first diagnostic criteria for bleeding derived via expert consensus applicable to critically ill children. It must be further validated, both in larger patient cohorts and in relation to clinical outcomes, in order to be applied in clinical trials.

## Conclusions and Future Directions

Pediatric intensivists will encounter bleeding on a relatively frequent basis in their patient population. In critically ill children, bleeding has been associated with significant morbidity and mortality. There is paucity of data on CRB acquired in PICU patients, stemming in part from the lack of a standardized pediatric bleeding score. The BASIC definition should be validated to clinical outcomes. This will facilitate the accurate identification of subpopulations of critically ill children at risk for CRB. The data generated from large scale epidemiologic studies can be used to development a numerical bleeding score which includes patient characteristics and laboratory values. This can be used, in turn, to plan monitoring strategies and prevention measures. A standardized and well-validated score can be used to define inclusion criteria or outcome and will aid in the development of large multicenter clinical trials to test current hemostatic interventions, such as platelet or plasma transfusion thresholds in children who are prone to bleed, such as those treated with anticoagulation, those in DIC or supported on ECMO and test new and investigational therapies, such as anti-fibrinolytics, factors replacements, use of whole blood and cold stored platelets.

## Author Contributions

AA, OK, and MN conceived of the format of the review and contributed to its writing. All authors reviewed the manuscript and edited it prior to submission.

## Conflict of Interest

The authors declare that the research was conducted in the absence of any commercial or financial relationships that could be construed as a potential conflict of interest.
